# Glycerol-3-phosphate acyltransferase 2 expression modulates cell roughness and membrane permeability: An atomic force microscopy study

**DOI:** 10.1371/journal.pone.0189031

**Published:** 2017-12-06

**Authors:** Elizabeth R. Cattaneo, Eduardo D. Prieto, Maria B. Garcia-Fabiani, Mauro A. Montanaro, Herve Guillou, Maria R. Gonzalez-Baro

**Affiliations:** 1 Instituto de Investigaciones Bioquímicas de La Plata Rodolfo R. Brenner, Consejo Nacional de Investigaciones Científicas y Técnicas, Facultad de Ciencias Médicas, Universidad Nacional de La Plata, La Plata, Buenos Aires, Argentina; 2 Instituto de Investigaciones Fisicoquímicas Teóricas y Aplicadas (INIFTA), La Plata, Buenos Aires, Argentina; 3 Toxalim, Université de Toulouse, INRA, ENVT, INP-Purpan, UPS, Toulouse, France; LAAS-CNRS, FRANCE

## Abstract

In mammalian cells, *de novo* glycerolipid synthesis begins with the acylation of glycerol-3-phosphate, catalyzed by glycerol-3-phosphate acyltransferases (GPAT). GPAT2 is a mitochondrial isoform primarily expressed in testis under physiological conditions, and overexpressed in several types of cancers and cancer-derived human cell lines where its expression contributes to the tumor phenotype. Using gene silencing and atomic force microscopy, we studied the correlation between GPAT2 expression and cell surface topography, roughness and membrane permeability in MDA-MB-231 cells. In addition, we analyzed the glycerolipid composition by gas-liquid chromatography. GPAT2 expression altered the arachidonic acid content in glycerolipids, and the lack of GPAT2 seems to be partially compensated by the overexpression of another arachidonic-acid-metabolizing enzyme, AGPAT11. GPAT2 expressing cells exhibited a rougher topography and less membrane damage than GPAT2 silenced cells. Pore-like structures were present only in GPAT2 subexpressing cells, correlating with higher membrane damage evidenced by lactate dehydrogenase release. These GPAT2-induced changes are consistent with its proposed function as a tumor-promoting gene, and might be used as a phenotypic differentiation marker. AFM provides the basis for the identification and quantification of those changes, and demonstrates the utility of this technique in the study of cancer cell biology.

## Introduction

Glycerol-3-phosphate acyltransferase (GPAT) catalyzes the first and committed step in the *de novo* glycerolipid synthesis pathway, which is the synthesis of lysophosphatidic acid (LPA) via the acylation of glycerol 3-phosphate by a long-chain fatty acyl-CoA substrate. Then, 1-acylglycerol-3-phosphate acyltransferase (AGPAT) uses LPA to form phosphatidic acid, the precursor for both triacylglycerol (TAG) and glycerophospholipid (PL) biosynthesis. In mammals, four GPAT isoforms (GPAT1–GPAT4) have been described which differ in their subcellular locations, tissue expression pattern, substrate preference, transcriptional regulation and sensitivity to sulfhydryl group reagents such as *N*-ethylmaleimide [1].

In physiological conditions, GPAT2 isoform resides in the outer mitochondrial membrane of primary spermatocytes and it is differentially expressed in the testis during sexual development [2,3]. GPAT2’s preferred substrate is arachidonoyl-CoA and this fatty acid (FA) is used to synthesize TAG *de novo* [2]. Because high levels of arachidonic acid (Δ^5,8,11,14^ eicosatetraenoic acid, 20:4 ω-6, AA) induce apoptosis [4–6], and metabolic pathways that diminish the content of unesterified AA can prevent apoptosis [7], enhanced GPAT2 activity may allow spermatogenic cells to sequester AA into TAG, a function that may be related to cell survival and proliferation [2,3].

In pathological conditions, we have reported that human GPAT2 is overexpressed in several types of cancers and cancer-derived human cell lines, and that its expression contributes to the tumor phenotype. In this regard, tumor cells with diminished GPAT2 expression had lower rates of cellular proliferation and migration and lower tumorigenicity in mouse xenograft models. In addition, we have shown that *GPAT2* belongs to a group of genes termed ‘cancer-testis genes’ (CTs) [8]. Proteins encoded by CTs are expressed in spermatogenic cells, whereas in somatic tissues their expression is either low or null. CTs are ectopically overexpressed in cancers of different origins where they may contribute to the tumor phenotype [9,10].

Cancer cells differ from normal cells in morphology, cell growth and migration rate, cell–cell interaction, cytoskeleton organization, and interactions with the extracellular matrix. Atomic force microscopy (AFM) is capable of detecting most of these changes [11]. AFM is used to scan surfaces at the nanometer (molecular) resolution scale, and it has emerged as a powerful tool to study the morphological and biomechanical properties of biological samples, including biomolecules and cells. This technique is appropriate for directly studying biological materials in buffer solutions or in fixed conditions. It allows sample observation in non-vacuous environments, without the need for coating, staining or freezing the material, and the resolution is similar to electron microscopy [12,13]. During the last few years, AFM has been increasingly used in biomedical research. It has been applied for the nanomechanical study of live cancer cells isolated from human metastatic fluids [14,15] and breast cancer tissue sections from different histological grades [16].

In this work, we used AFM to evaluate the phenotypic consequence of *GPAT2* expression in cancer cells, and to correlate human *GPAT2* expression with the cellular processes that exacerbate the tumoral phenotype in a breast cancer cell model.

## Materials and methods

All chemicals were purchased from Sigma unless otherwise indicated.

### Cell line and culture conditions

Human breast adenocarcinoma MDA-MB-231 cells were purchased from the American Type Culture Collection (ATCC, Manassas, VA, USA). Cells were routinely cultured in Dulbecco’s modified Eagle’s medium (DMEM, Gibco) supplemented with 10% FBS (Natocor, Argentina), 100 U/ml penicillin, 100 μg/ml streptomycin and 2 mM glutamine. Cells were grown at 37°C in a 5% CO_2_ atmosphere with 98% relative humidity. We chose the MDA-MB-231 cells because of its high *GPAT2* expression.

### MDA-MB-231 *GPAT2* silencing

Cell lines stably expressing a small-hairpin RNA targeting *GPAT2* mRNA (shRNA-GPAT2) or a non-silencing scrambled RNA (shRNA-scr) were developed in our laboratory from commercial MDA-MB-231 cells, as previously reported [8] to generate sh-MDA (reduced *GPAT2* expression) and scr-MDA (retaining *GPAT2* expression) cell lines. *GPAT2* knockdown was routinely assessed by quantitative real-time PCR (qRT-PCR) [8] and Western blot.

### Quantitative real-time PCR

Total RNA was isolated from cell lines using TRIZOL (Life Technologies) following the manufacturer’s instructions, and RNA quality was determined by gel electrophoresis and 260/230 and 260/280 nm absorbance ratios. One μg RNA was used for cDNA synthesis employing iScript cDNA synthesis Kit (Bio-Rad). A 1:10 cDNA dilution was used for the qRT-PCR reaction with iTaq Universal Sybr Green Super Mix (Bio-Rad). Primers were designed to amplify:

Human GPAT2

forward primer: AAG CTG GTG TGA GGT GAG AG

reverse primer: ATA CTT CCC CAG GAA TGG AG

AGPAT11/LPCAT2

forward primer: ATA GCC CAA GGG GAC TCA AT

reverse primer: GAA AAC ACA TGG CAC GTC TG

MBOAT5/LPCAT3

forward primer: GCG GCT GAT CAT CTC CAT CTT

reverse primer: TGG TAG AGC TGG TTT CCA AAG

MBOAT7/LPIAT1

forward primer: CTC AGC TCT CCG TTC TCG AC

reverse primer: GAA CAG ACG GGC TCT GGA AA

The thermal profile was 95°C for 1 min, followed by 45 cycles of 95°C for 20 s, 57°C for 50 s and 60°C for 30 s, on a Stratagene Mx3000P apparatus. RNA expression was quantified in triplicate using the ΔΔCt method, and normalized to that of TBP housekeeping gene using Qbase software.

### Immunoblotting

One-hundred μg of total protein from cells was separated on 10% SDS-PAGE, transferred to a polyvinylidene difluoride membrane (BioRad) and probed with 1/1000 anti-GPAT2 antibody (Sigma HPA036841). Anti-β-actin antibody (Abcam ab8227) at a dilution 1:2500 was used as a gel-loading control. Membranes were then washed extensively and probed with horseradish peroxidase-conjugated goat anti- rabbit or anti- mouse IgG antibody (Thermo-Pierce). For chemiluminescent detection, the membranes were incubated with Super Signal detection kit (Thermo-Pierce).

### Lipid and fatty acid analysis

scr-MDA and sh-MDA cells were grown in 100 mm plates in routine medium or in routine medium supplemented with 50 μM AA as the sodium salt (Sigma) for 72 h. Cells were then washed 3 times with 0.1% BSA in ice-cold PBS, and scraped in ice-cold methanol and H_2_O. Total lipids were extracted [17] and separated by TLC on silica-gel G60 plates (Merck) with hexane-ethylether-acetic acid (80:20:1; v/v/v) as the mobile phase for neutral lipid separation. All samples were chromatographed in parallel with pure lipid standards. To analyze the FA composition, total PL and TAG fractions were scraped from the plate and eluted with hexane: chloroform: methanol (3:2:1, v/v/v). FA methyl esters were obtained by reaction with BF_3_ in methanol and analyzed by gas liquid chromatography in a Hewlett-Packard HP 6890 chromatograph equipped with an Omega Wax capillary column [2].

### Optical and fluorescence microscopy

Live cell images were captured with an inverted microscope (Olympus, IX71) equipped with a fluorescent lamp and a digital camera (Olympus) under 200-x magnification.

### Atomic force microscopy

For Atomic force microscopy (AFM) measurements, scr-MDA (control) and sh-MDA (*GPAT2* silenced) cells were plated on glass coverslips and grown in routine medium without the addition of AA for 1−2 days until 80% confluent. Then, the cells were fixed using an ethanol dehydration train at room temperature and subsequently air dried [18]. Fixation is necessary because high-resolution images of cells are poorly achieved in solution, and the enhanced AFM resolution obtained with fixed cells allows a more detailed subcellular analysis [12].

AFM images were obtained in air, using a MultiMode Scanning Probe Microscope (Veeco) equipped with a Nanoscope V controller (Veeco). All measurements were obtained immediately after fixation with Tapping^®^ mode, using probes doped with silicon nitride (RTESP, Veeco with tip nominal radius of 8–12 nm, 271–311 kHz, force constant 20–80 N/m). Typical scan rates were 0.5 Hz.

The analysis was first performed on a large area (50 μm^2^) with a single cell, and later by heading the cantilever to the cell surface to obtain images of 30 μm^2^, 15 μm^2^, and 10 μm^2^. The same procedure was repeated in six different cells (three cells each time in two independent AFM measurements), with three different AFM acquisition modes: height, phase, and amplitude.

### Roughness analysis

The effect of *GPAT2* silencing on membrane roughness of MDA-MB-231 cells was determined using AFM and taking into consideration the Ra and Rq values as quantitative parameters. The parameter Ra is the arithmetic mean of the deviations in height from the roughness mean value, and Rq is the root mean square of the height distribution. In the current bibliography, the minimum number of cells necessary to perform roughness studies is not standardized. Thus, some authors analyze three [19], four [16] or more cells. Here, for the analysis, six different cells from each group were examined and four different areas from each cell were used to calculate the Ra and Rq. The selected areas were 5 x 5 μm^2^. They were above the cytoplasmic region, and outside of the pore zone for sh-MDA cells, on images of 15 x 15 μm^2^. AFM image and data analysis was performed using the Nanoscope 7.30 and Nanoscope Analys 1.5 software package.

### Lactate dehydrogenase activity

To evaluate plasma membrane integrity, cells were grown in a 48-well microplate, and a commercial kit by the decrease of OD at 340 nm (Wiener lab) was used to measure the release of the cytoplasmic enzyme lactate dehydrogenase (LDH).

### Statistical analysis

Differences between the control and silenced cells were analyzed by Student’s t-test or ANOVA-Tukey test. Results were considered significant at the 5% level. Statistical comparisons were performed with InStat3 software.

## Results and discussion

### MDA-MB-231 cells compensate for the lack of GPAT2 by overexpressing the acyltransferase AGPAT11

**Fig 1:**
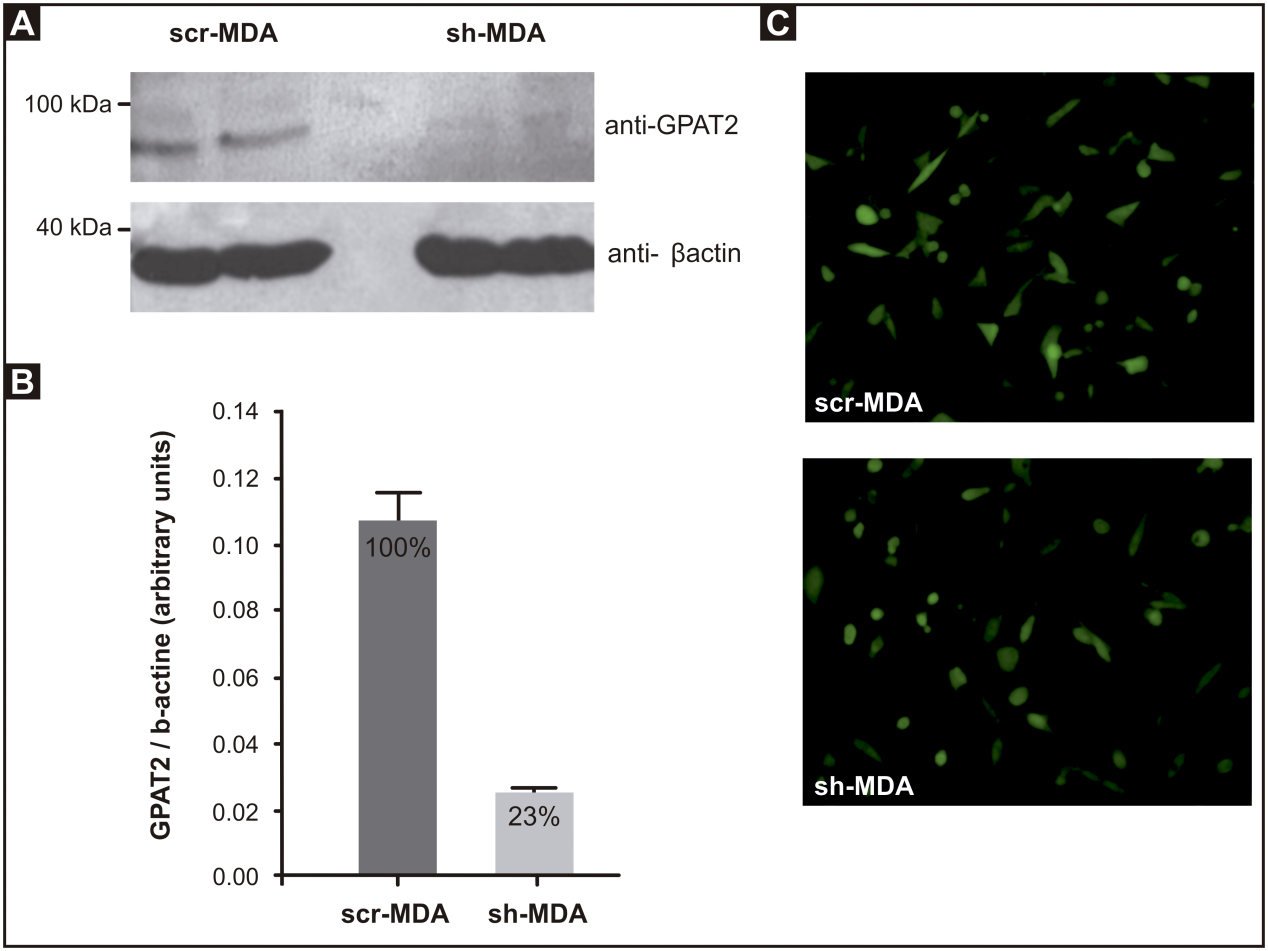
*GPAT2* knock-down in MDA-MB-231 cells. MDA-MB-231 cells were silenced for human *GPAT2* with HuSH-29 plasmid coding for a shRNA against human *GPAT2* mRNA (sh-MDA). A non-effective scrambled sequence shRNA plasmid was used to create a negative control (scr-MDA). **A)** Protein expression was measured by Western blot. Total cell lysate (100 μg protein) from scr-MDA and sh-MDA cells was probed with anti-GPAT2 antibody and with anti-β-actin antibody as a loading control. **B)** Protein band intensities were quantified with the ImageJ program. Results are representative of three independent experiments (** p< 0.01). **C)** Fluorescence image showing GFP expression in scr-MDA and sh-MDA at 200x magnification confirm that cells were transfected.

We used RNA interference to knock-down *GPAT2* expression in MDA-MB-231 cells and obtained 2 stable cell lines: sh-MDA and its control, scr-MDA. Compared to scr-MDA cells, a 95% down-regulation of *GPAT2* mRNA was obtained in sh-MDA cells [8]. In this work, the GPAT2 knockdown was confirmed by Western blot, using an antibody against GPAT2 (Fig 1A and 1B).

Previously, we reported that GPAT2 specifically uses AA as a substrate [2]. For the present study, our aim was to determine the role of the human GPAT2 isoform in glycerolipid metabolism and plasma membrane functionality in a cancer cell model.

**Fig 2:**
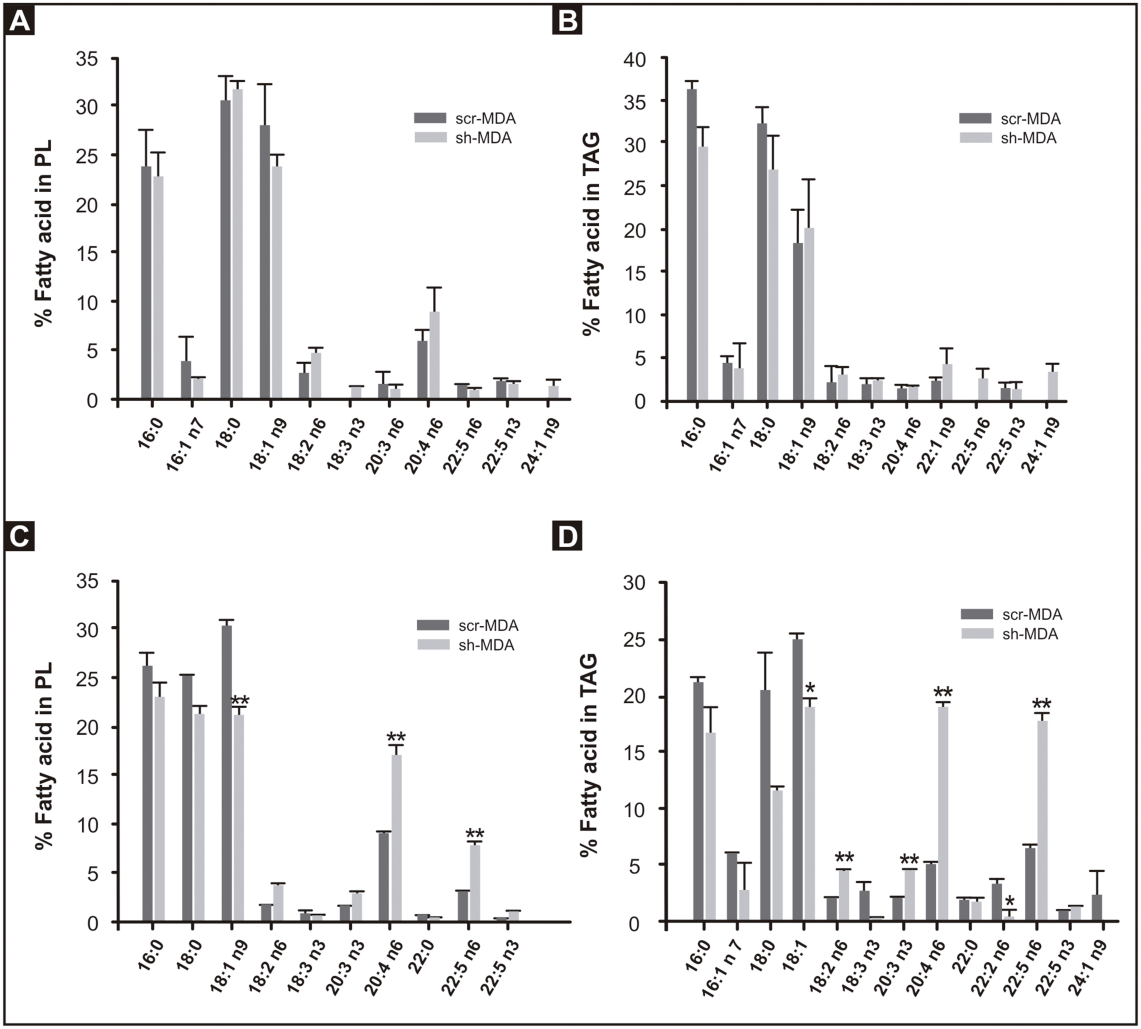
Fatty acid composition of scr-MDA and sh-MDA cell PL and TAG. Total lipids were extracted from scr-MDA and sh-MDA cells, separated by TLC, and fatty acid composition of the PL and TAG fractions was determined by GLC. **A and B)** Cells grown in DMEM 10% FBS. **C and D)** Cells grown in DMEM 10% FBS plus 50 μM of AA for 3 days. Values represent the mean ± SD of 3 independent experiments (* p<0.05, ** p< 0.01).

To do this, we isolated the PL and TAG fractions from scr-MDA and sh-MDA cells grown in routine medium and analyzed the FA compositions (Fig 2A and 2B). Surprisingly, no significant differences were observed in the AA content of TAG and PL in either cell line. Because the concentration of AA in the media was low and to expose the cells to a higher amount of AA, we analyzed the FA composition of PL and TAG fractions of cells, grown for 3 days in DMEM plus 50 μM AA (Fig 2C and 2D). We chose this AA concentration because incubation of MDA-MB-231 cells with 200 μM AA for 72 h does not affect cell viability [20], but sh-MDA cells were more vulnerable and died when exposed to 100μM AA. In the presence of 50 μM AA for 3 days, sh-MDA cells incorporated more than twice as much AA than scr-MDA cells in both PL (17.2 vs 8.9%; p< 0.01) and TAG (19.0 vs 5.1%; p< 0.01). The 22:5n6 product of AA was similarly increased in glycerolipids in the supplemented cells.

**Fig 3:**
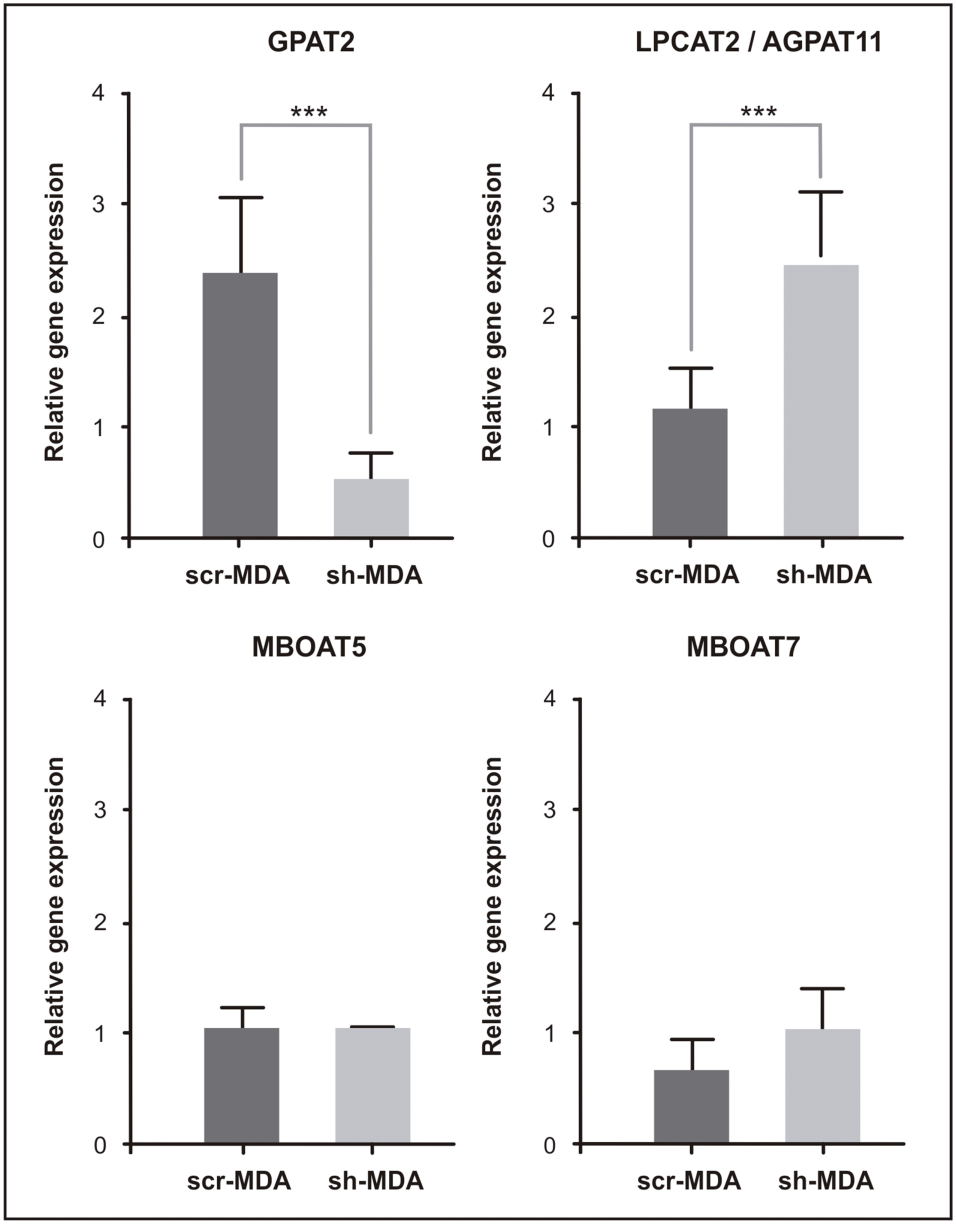
mRNA expression of acyltransferases involved in arachidonic acid metabolism. Total RNA from scr-MDA and sh-MDA cells was extracted, subjected to cDNA synthesis, and amplified by qRT-PCR with primers for human *GPAT2*, *AGPAT11*, *MBOAT5* and *MBOAT7* genes and normalizing expression levels to that of *TATA-Box binding protein (TBP)* housekeeping gene (*** p<0.001. ANOVA-Tukey test).

AA is an essential FA that is obtained directly from the diet, or indirectly synthesized from linoleic acid. AA plays important roles in cell metabolism. The bulk of AA in mammalian cells is rapidly incorporated into cell membrane phospholipids. In addition to its use as a substrate for glycerolipid synthesis, free AA may exert signaling functions as an inducer of apoptosis or as an eicosanoid precursor. Because eicosanoids exert potent biological actions, cells keep AA at very low levels by promoting their esterification into cell lipids [21]. Taking into account the biological importance of AA, we suspected that to compensate the lack of GPAT2, sh-MDA cells might overexpress other enzymes capable of incorporating AA into glycerolipids. To test this hypothesis we measured the expression of other three enzymes involved in the glycerolipid metabolism: AGPAT11/LPCAT2, MBOAT5/LPCAT3 and MBOAT7/LPIAT1. These three enzymes are able to use arachidonoyl-CoA as a substrate [21–24]. 1-acyl-glycerol-3-phosphate *O*-acyltransferase (AGPAT) and membrane bound *O*-acyltransferase (MBOAT) are two families of lipid acyltransferases. The MBOAT family is comprised of members specifically involved in the Lands cycle of phospholipid FA remodeling, whereas members of the AGPAT family are typically involved in the pathway *of de novo* glycerolipid biosynthesis. qRT-PCR analysis showed that compared to scr-MDA cells, *AGPAT11* expression in sh-MDA cells was 2-fold higher. The expression levels of *MBOAT5* and *MBOAT7* were equivalent in both cell lines (Fig 3).

GPAT2 specifically uses AA as a substrate [2], and *GPAT2* silencing increased the expression of another glycerolipid biosynthetic enzyme that prefers AA, AGPAT11 (Fig 3). AGPAT11 uses arachidonoyl-CoA as a substrate for both its 1-acylglycerol-3-phosphate-O-acyltransferase and lysophosphatidylcholine acyltransferase activities [24]. Because GPAT isoforms catalyze the first and rate-limiting step in *de novo* glycerolipid biosynthesis, we propose that AGPAT11 overexpression compensated for the decrease of GPAT2 and may be partially responsible for the higher AA content of the glycerolipids after GPAT2 silencing. AA incorporation into glycerolipids depends on its concentration (high or low affinity pathways), the cell type, and the activity of many different enzymes [21], so we cannot exclude the possibility that other enzymes may be also be important in determining the overall composition of the cell glycerolipids in both culture conditions.

### Cells with deficient GPAT2 exhibit smoother topography and pore-like structures

The plasma membrane is involved in several cellular functions, including proliferation, signal transduction, motility, and differentiation. Because *GPAT2* silencing alters cell proliferation and migration [8], we decided to study the topography and plasma membrane functionality in our cell model. For this, we used AFM technique. AFM provides a tri-dimensional mapping of the cell surface, resulting in the generation of true topographic data with vertical resolution into the subnanometer range. To minimize the tip impact onto the cell surface we used a dynamic or intermittent contact mode, commonly known as Tapping mode. Tapping mode is characterized for the rapid up-and-down oscillation of the tip, which slowly scans the sample laterally and touches the surface for very short periods of time [13]. Because AFM images may be affected by the geometrical shape of the probe [25,26], and to minimize the artifacts in cell imaging and mechanical measurements, conventional AFM pyramidal tips are routinely used [27]; so we used pyramidal tips for all our measurements.

Since AFM is a novel tool to observe biological samples, one of its difficulties is the lack of optimized protocols, mainly for the pre-analytical phase of sample preparation, which makes it difficult to compare results between laboratories. In this sense, it have been reported that the measurements of roughness and viscoelasticity, as well as the cell morphology, are affected depending on the fixation method, suggesting the necessity of an standard fixation method, applicable to various cell types [28,29]. Also, the fixation procedure may introduce artifacts such as cell debris, crystals (commonly derived from the washing buffer, PBS) or depressions [30]. In addition, for many of the commercially available cell lines there are no previous data of AFM topography studies. Fortunately, the structure of MDA-MB-231 cells has previously been elucidated using AFM [31], so we decided to use the same fixation conditions. This fixation method preserves the epithelial morphology of MDA-MB-231 cells, and during the acquisition of our images, we did not detect any of the most common fixation artifacts reported in the bibliography [30].

#### Surface nanostructure imaging (Cell topography)

**Fig 4:**
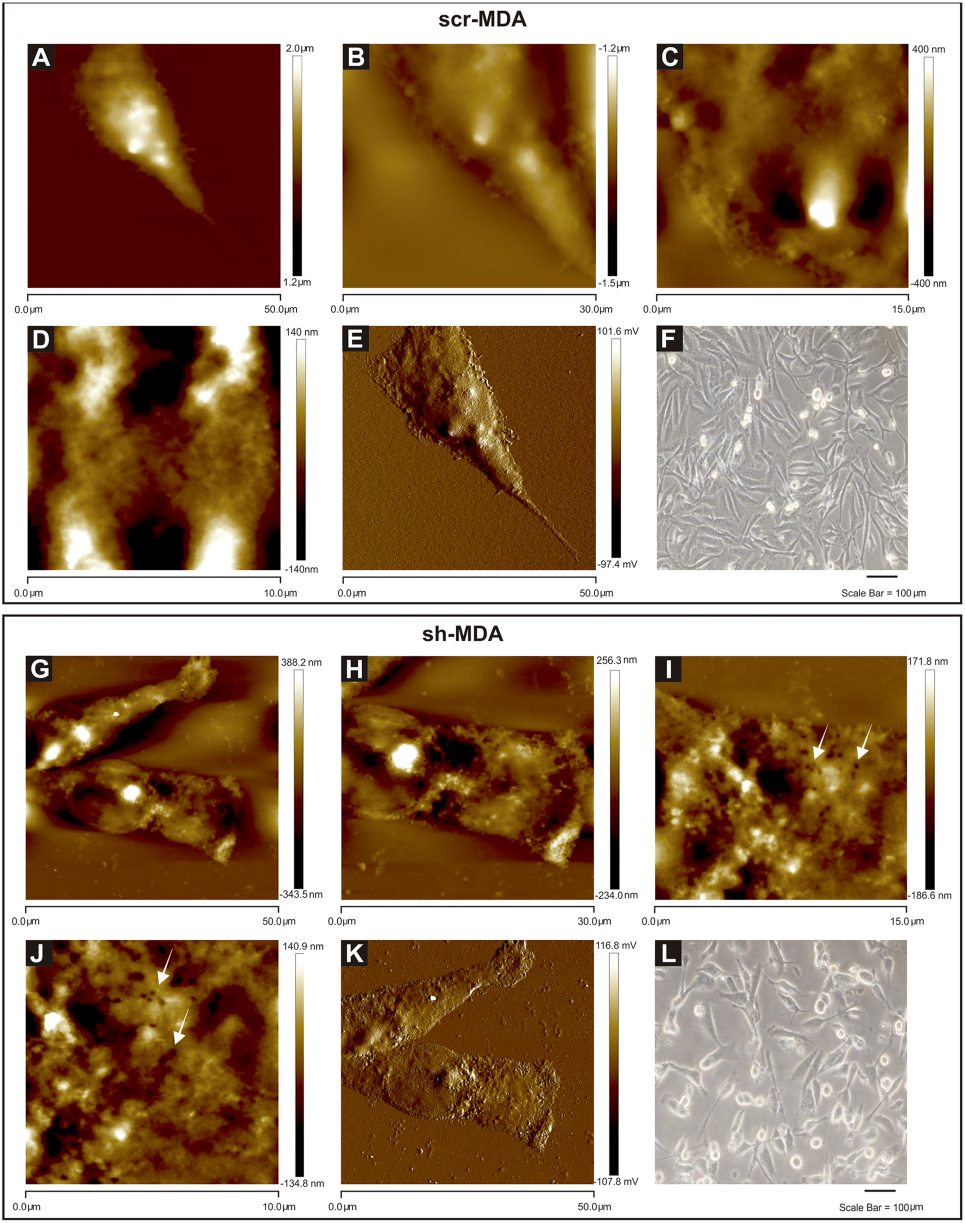
Optical microscopy and AFM images of scr-MDA and sh-MDA cells. AFM images were obtained in tapping topography (A-D for scr-MDA and G-J for sh-MDA) and amplitude modes (E for scr-MDA and K for sh-MDA). Fig 4A presents topographical parameters of a complete scr-MDA cell. Fig 4E presents amplitude image. Fig 4G presents topographical parameters of complete sh-MDA cell, and Fig 4K presents amplitude image. Parameters of the pictures are 50.0 μm x 50.0 μm (Fig 4A, E, G and K); 30.0 μm x 30.0 μm (Fig 4B and H); 15.0 μm x 15.0 μm (Fig 4C and I); 10.0 μm x 10.0 μm (Fig 4D and J). All images have a resolution of 512 x 512 pixels. The height of the cell is expressed in color scale (right bar). White arrows in Figs 4I and J indicate pore-like structures. Optical images (Fig 4F and L) have a magnification of 200x.

To determine the phenotypic differences between *GPAT2* expressing and silenced cells we analyzed two types of images from AFM: topography and amplitude images (Fig 4). Topography images show the contour heights, while amplitude images show how the tip deflected as it encountered simple topography.

Direct comparison of scr-MDA and sh-MDA cells (Fig 4C and 4D (scr-MDA) and Fig 4I and 4J (sh-MDA)) revealed marked differences in surface morphology at micro- and nanoscale. The most notable was the presence of pore-like structures in the sh-MDA cells (white arrow, Fig 4I and 4J).

Comparing optical microscopy (Fig 4F and 4L) with AFM (Fig 4A and 4G) images, we observe that the fixed cell morphology is consistent with that of live cells.

Cell surface imaging was earlier considered as an important biological application of the scanning probe microscopy and, more particularly, of the AFM. Thus, these observations demonstrate the uniqueness of AFM for detecting micro- to nanoscale differences for the morphological characterization of mammalian cell surfaces.

#### Roughness

**Fig 5:**
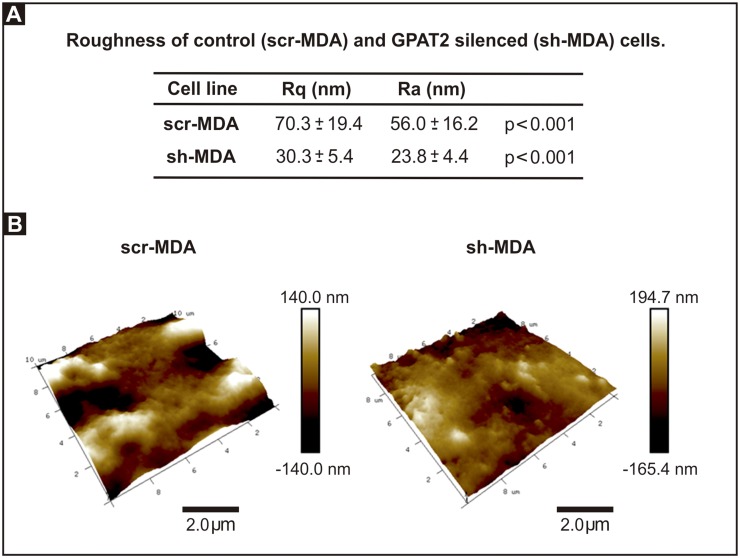
Cells sub-expressing GPAT2 exhibit smoother topography. **A)** Ra and Rq values of scr-MDA and sh-MDA cells. **B)** 3D AFM topography images of scr-MDA and sh-MDA cells at 10.0 μm x 10.0 μm showing cell surface details.

The topography images also provided information about the details of the cell surface structure and its smoothness or roughness. Because roughness value depends on the size of the sampling field, we analyzed all images in areas of a fixed size of 5 × 5 μm^2^ [23], above the cytoplasmic region, on topography images of 15 x 15 μm^2^; and we determine that Ra and Rq, measures of surface roughness, both decreased significantly in sh-MDA cells. Comparing the Rq values, *GPAT2* silencing decreased roughness by 57% (Fig 5). To discard the possibility that the difference in roughness was due to the fixation method we repeated the roughness analysis using cells fixed for 15 minutes with 2.5% paraformaldehyde in PBS and we obtained equivalent results (*GPAT2* silencing decreased Rq by 36%).

Surface roughness analysis demonstrated measurable differences between *GPAT2*-silenced and control cells, providing novel quantifiable data of cellular changes related to *GPAT2* expression at the cell surface level.

#### Membrane integrity

**Fig 6:**
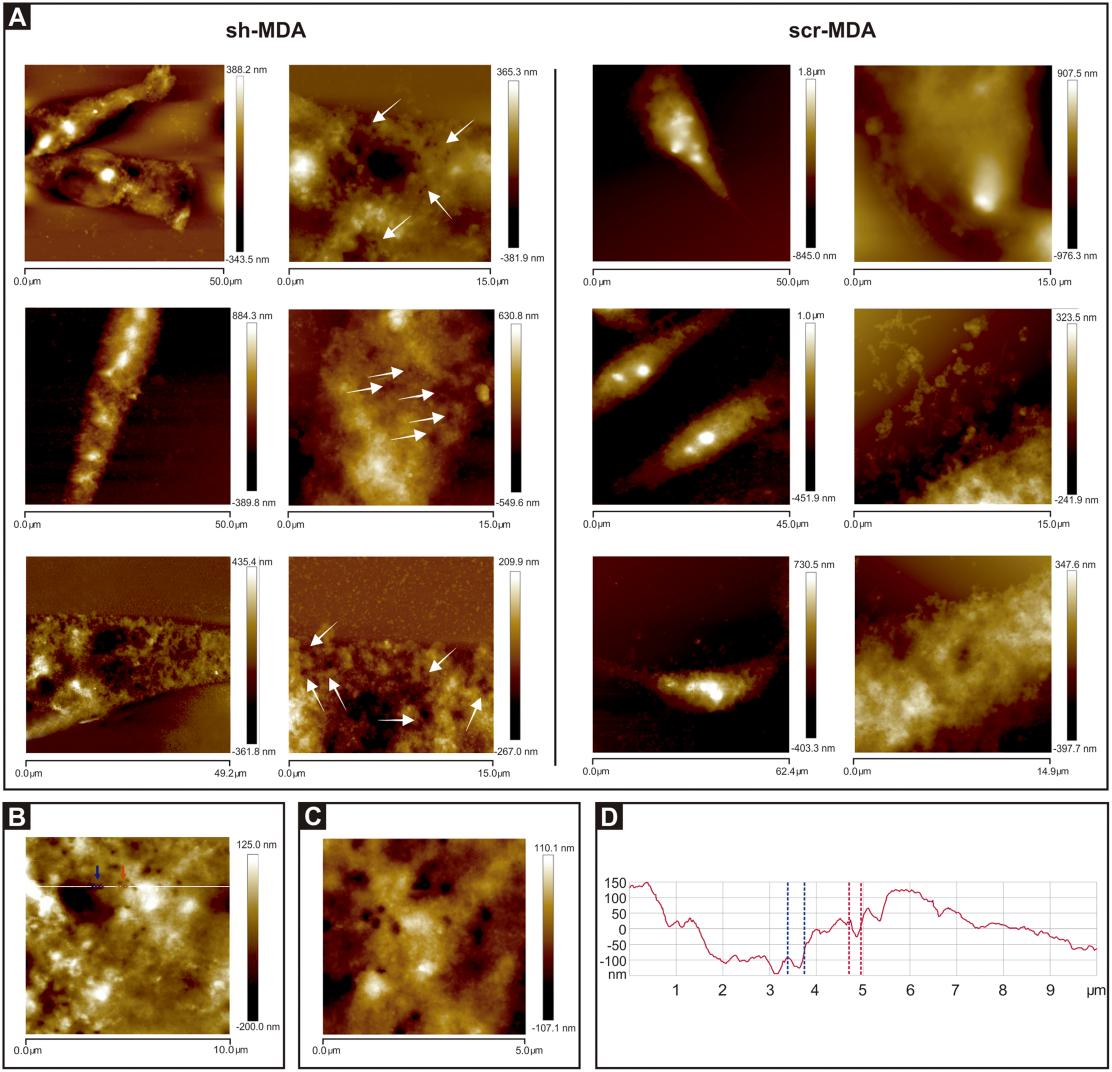
Cells sub-expressing *GPAT2* exhibit pore-like structures. **A) Representative** AFM images of three different sh-MDA and scr-MDA cells revealed pore-like structures only on the surface of sh-MDA cells indicated with white arrows in 15.0 μm x 15.0 μm images. **B and C)** High-resolution 10.0 μm x 10.0 μm and 5.0 μm x 5.0 μm images, respectively, of one sh-MDA cell showing pore-like structures. **D)** Section analysis showing the size of two pores along the white line shown in the Fig 6B. The dashed red and blue lines define the diameter and depth of the pores indicated with red and blue arrows, respectively, in Fig 6B. Fig 6B is the section analysis of the cell shown in Fig 4J.

High-resolution AFM imaging revealed membrane damage, visualized as pore-like structures randomly distributed on the surface of sh-MDA cells (white arrows Fig 6A). The amount of pore-like structures observed in sh-MDA cells was 0.56 ± 0.11 pore-like structures/ μm^2^. We performed the section analysis of our images of sh-MDA cells and the average pore size diameter was 270.4 ± 52.1 nm (n = 21). In none of the scr-MDA images (Fig 6A) pore-like structures were observed.

**Fig 7:**
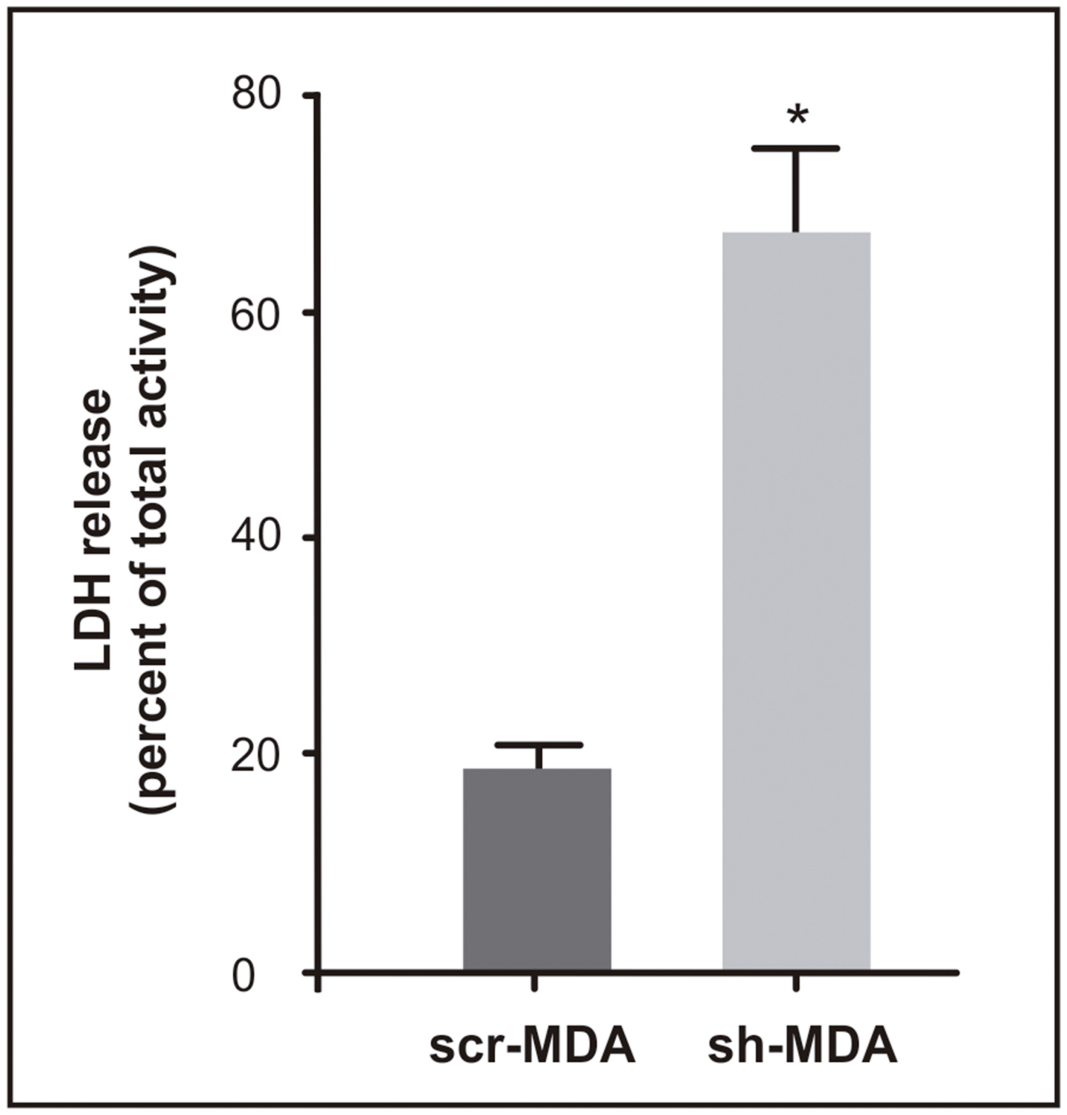
*GPAT2* silencing disrupts membrane integrity. Cells were grown in a 48-well microplate and LDH activity was determined in the cellular supernatants and in total cell lysates using a commercial colorimetric method. Results are expressed as the percentage of LDH activity in the supernatants for each cell line. Results are the means ± SD of 3 independent experiments. (* p<0.05)

AFM has been used to study pore formation in other biological systems [19,32,33]. However, AFM technique is a single-cell assay so; to validate this result and to analyze the biological outcome of the pore presence we quantified LDH activity in the cellular supernatants of both cell lines. sh-MDA released 3.6-fold more LDH to the medium than scr-MDA cells (Fig 7). LDH is a cytosolic enzyme, its presence in the culture medium indicates membrane damage and is associated with less healthy cells [34,35]. This result correlates with our previously reported diminished cell proliferation rate for sh-MDA cells [8].

*GPAT2* is a cancer-testis gene (http://www.cta.lncc.br/). CTs are normally expressed in germ line cells but they are also activated in a wide range of cancer types, where they usually encode antigens that are immunogenic and present a potential use as biomarkers and targets for immunotherapy. Most of the CTs’ functions have not been elucidated yet, however, it was recently reported that they may have a role in the regulation and progression of the cell cycle, the control of transcription, cell survival and apoptosis [36].

In a previous work, we showed that *GPAT2* expression increased cell proliferation and cell migration rate, and resistance to staurosporine-induced apoptosis [8]. All these features are related to the normal plasma membrane functionality, and for this reason in this work we studied for the first time the topography and membrane properties of human breast cancer-derived cells that express GPAT2 (scr-MDA), and compared with cells with silenced GPAT2 (sh-MDA).

We chose the MDA-MB-231 because it is the cell line with the highest *GPAT2* expression [8], and we found that although *GPAT2* silencing did not produce significant changes in total lipid membrane composition, it modified parameters directly related to the plasma membrane functionality, such as roughness and permeability.

In part, this did not surprise us because it was previously reported that cell topography and mechanical properties can change without major alterations in total lipid composition, as occurs in HeLa cells at different stages of the cell cycle [37].

Nevertheless, our observation that cells expressing *GPAT2* exhibit a rougher topography and do not have pore-like structures compared to *GPAT2* silenced cells, directly implicate GPAT2 in the regulation of these cell-surface characteristics.

Surface roughness is an established method for quantitative surface analysis. Cellular health can be strongly implied by plasma membrane roughness, and AFM-Rq analysis is useful for determining the signaling molecule effect on cells [19], gene silencing or overexpression [16], and malignant transformation [11,38]. Several studies have shown that membrane roughness increments correlate with tumor phenotype exacerbation [11]. In particular, for breast cancer, it was observed that compared to benign breast MCF-10 cells, the cancerous cells MCF-7 exhibit a more disorganized filamentous cytoskeleton structure with rougher membranes [38]. In addition, in human breast cancer histological sections, surface roughness increases in accordance with cancer grade [16].

In this work, we found that control cells were 2.3-fold rougher than *GPAT2* silenced cells. Thus, our roughness measurements agree with those previously reported and with our previous results showing that *GPAT2* expression contributes to the tumor phenotype of MDA-MB-231 cells [8].

Surface roughness has also been used as a tool for investigating cytoskeleton structure and integrity [39–41]. The cytoskeleton framework is a dynamic structure mainly composed of microfilaments, microtubules and intermediate filaments. The activities of many proteins are deregulated in cancerous cells, and that includes the proteins involved in the dynamic reorganization of the cytoskeleton, which alter cell motility, morphology, adhesion and invasion [16]. Since the underlying cytoskeleton framework governs the cellular morphology, several studies have shown that membrane roughness variations are induced by cytoskeleton alterations. Those alterations were observed in both cytoskeleton structural and remodeling proteins [11,40–43], and in non-cytoskeletal-related proteins, such as SMAR 1 [16]. The tumor suppressor protein SMAR 1 expression alters the cellular cytoskeleton, decreasing the roughness of the cell membranes [16].

Like SMAR 1, GPAT2 is not directly related to cytoskeleton composition or remodeling.

Then, to explain the cell roughness modifications associated with GPAT2 presence, we decided to study the expression of cytoskeleton and cytoskeleton-associated proteins in control and sh-MDA cells. For this, we analyzed the results of a transcriptomic experiment performed by our group in scr-MDA and sh-MDA cells using an Agilent SurePrint G3 Human Gene Expression 8x60K v2 Microarray (S1 File). After filtering off the data for a |Log Fold Change| >2 and a p value<0.01 we observed that, compared to the control cells, some genes encoding for proteins involved in the regulation of cellular cytoskeleton were upregulated or downregulated in cells subexpressing GPAT2 (Table 1).

**Table 1:** Cytoskeleton related genes upregulated or downregulated in sh-MDA cells.

Gene Symbol	Name	Up/down in sh-MDA	Log FC sh-scr
TUBB2B	tubulin beta 2B class IIb	Upregulated	2.40
TPPP	tubulin polymerization promoting protein	Upregulated	2.28
AFAP1-L1	actin filament associated protein 1-like 1	Downregulated	- 4.31
ARP-M1	actin related protein M1	Downregulated	- 2.57

These results imply that the decrease in roughness in *GPAT2* subexpressing cells could be due to the alterations in the expression of cytoskeleton and cytoskeleton remodeling proteins, but more detailed studies will be necessary to correlate *GPAT2* expression with cytoskeleton function and remodeling.

On the other hand, the AFM resolution is able to reveal the presence of micro-irregularities such as pore-like structures. In this sense, pore-like structures were studied at the apical membrane of pancreatic acinar cells [32], in the outer mitochondrial membrane [33], and at the rituximab induced lysis of lymphoma cells [44]. Thus, this technique has been used to study pore presence or formation in both physiological and non-physiological conditions.

Membrane integrity is critical to cell survival and function, and a reduction in cell viability induced by pore formation is commonly observed in pathological conditions such as drug treated, malignant, or virally transformed cells [44]. Here, we observed that only sh-MDA cells exhibited pore-like structures and they were not present in *GPAT2* expressing cells. We discard that pore-like structures are a consequence of the fixation procedure because first, both cell lines were fixed and AFM scanned under the same optimal conditions, being the pore-like structures present exclusively in sh-MDA cells; and second, only sh-MDA cells present physiological alterations compatible with membrane damage. The sh-MDA cells show a higher release of LDH to the medium and a lower proliferation rate, compared to control cells. Moreover, the disturbance in the cytoskeleston integrity can be linked to the formation of pores. In this sense, AFM analysis showed that the treatment of HeLa cells with colchicine, a microtubule-depolymerazing drug, produced pores in the cell membrane [42]. Therefore, these results suggest that *GPAT2* silencing alters the integrity of the cell membrane, leading to an increase in its permeability and a reduction in the cellular proliferation rate previously reported.

## Conclusion

AFM is a powerful tool for basic research in cancer biology and the characterization of this disease progression. In this sense, most of the recent studies have used AFM for exploratory research on cancer cell morphology, cell topography and mechanical properties, or the relationship of these to cell function.

In the present work, we report for the first time the morphological differences between cells that express or subexpress a cancer-testis gene *GPAT2* in a breast cancer cellular model. *GPAT2* silencing decreases cell roughness and induces the formation of pore-like structures, which correlates with an increment in cell membrane permeability. Cancer-testis genes have been proposed as targets for anticancer vaccines, however, the roles of these reactivated testis genes in supporting tumorigenic features have been less studied. Here, based on *GPAT2* expression, we validated AFM as a modern and valuable tool to correlate plasma membrane topographical alterations with malignant phenotype.

## Supporting information

S1 File Microarray transcriptomic data. scr-MDA and sh-MDA cell mRNA expression profile was analyzed using an Agilent SurePrint G3 Human Gene Expression 8x60K v2 Microarray.
(XLSX)

## Abbreviations

AA: arachidonic acid
AFM: atomic force microscopy
AGPAT: 1-acylglycerol-3-phosphate acyltransferase
CT: cancer-testis gene
FA: fatty acid
GLC: gas liquid chromatography
GPAT: glycerol-3-phosphate acyltransferase
LDH: Lactate dehydrogenase
LPA: lysophosphatidic acid
MBOAT: membrane bound *O*-acyltransferase
PL: glycerophospholipid
qRT-PCR: quantitative real-time PCR
scr-MDA: MDA-MB-231 that express *GPAT2*
sh-MDA: MDA-MB-231 cells with reduced *GPAT2* expression
TAG: triacylglycerol
TLC: thin layer chromatography

## References

[1] Wendel AA, Lewin TM, Coleman RA (2009) Glycerol-3-phosphate acyltransferases: rate limiting enzymes of triacylglycerol biosynthesis. Biochim Biophys Acta 1791: 501–506. doi: 10.1016/j.bbalip.2008.10.010 19038363 10.1016/j.bbalip.2008.10.010 PMC2737689

[2] Cattaneo ER, Pellon-Maison M, Rabassa ME, Lacunza E, Coleman RA, Gonzalez-Baro MR (2012) Glycerol-3-phosphate acyltransferase-2 is expressed in spermatic germ cells and incorporates arachidonic Acid into triacylglycerols. PLoS One 7: e42986 doi: 10.1371/journal.pone.0042986 22905194 10.1371/journal.pone.0042986 PMC3414494

[3] Garcia-Fabiani MB, Montanaro MA, Lacunza E, Cattaneo ER, Coleman RA, Pellon-Maison M, et al (2015) Methylation of the Gpat2 promoter regulates transient expression during mouse spermatogenesis. Biochem J 471: 211–220. doi: 10.1042/BJ20150730 26268560 10.1042/BJ20150730 PMC4613502

[4] Fonteh AN, LaPorte T, Swan D, McAlexander MA (2001) A decrease in remodeling accounts for the accumulation of arachidonic acid in murine mast cells undergoing apoptosis. J Biol Chem 276: 1439–1449. doi: 10.1074/jbc.M006551200 11022038 10.1074/jbc.M006551200

[5] Perez R, Matabosch X, Llebaria A, Balboa MA, Balsinde J (2006) Blockade of arachidonic acid incorporation into phospholipids induces apoptosis in U937 promonocytic cells. J Lipid Res 47: 484–491. doi: 10.1194/jlr.M500397-JLR200 16326977 10.1194/jlr.M500397-JLR200

[6] Wolf LA, Laster SM (1999) Characterization of arachidonic acid-induced apoptosis. Cell Biochem Biophys 30: 353–368. doi: 10.1007/BF02738119 10403056 10.1007/BF02738119

[7] Cao Y, Pearman AT, Zimmerman GA, McIntyre TM, Prescott SM (2000) Intracellular unesterified arachidonic acid signals apoptosis. Proc Natl Acad Sci U S A 97: 11280–11285. doi: 10.1073/pnas.200367597 11005842 10.1073/pnas.200367597 PMC17191

[8] Pellon-Maison M, Montanaro MA, Lacunza E, Garcia-Fabiani MB, Soler-Gerino MC, Cattaneo ER, et al (2014) Glycerol-3-phosphte acyltransferase 2 behaves as a cancer testis gene and promotes growth and tumorigenicity in the breast cancer MDA-MB-231 cell line. PLoS One 9: e100896 doi: 10.1371/journal.pone.0100896 24967918 10.1371/journal.pone.0100896 PMC4072688

[9] Hofmann O, Caballero OL, Stevenson BJ, Chen YT, Cohen T, Chua R, et al (2008) Genome-wide analysis of cancer/testis gene expression. Proc Natl Acad Sci U S A 105: 20422–20427. doi: 10.1073/pnas.0810777105 19088187 10.1073/pnas.0810777105 PMC2603434

[10] Whitehurst AW (2014) Cause and consequence of cancer/testis antigen activation in cancer. Annu Rev Pharmacol Toxicol 54:251–72. doi: 10.1146/annurev-pharmtox-011112-140326 Epub;%2013 Oct 11.: 251–272. 24160706 10.1146/annurev-pharmtox-011112-140326

[11] Canetta E, Riches A, Borger E, Herrington S, Dholakia K, Adya AK (2014) Discrimination of bladder cancer cells from normal urothelial cells with high specificity and sensitivity: combined application of atomic force microscopy and modulated Raman spectroscopy. Acta Biomater 10: 2043–2055. doi: 10.1016/j.actbio.2013.12.057 24406196 10.1016/j.actbio.2013.12.057

[12] Francis LW, Lewis PD, Wright CJ, Conlan RS (2009) Atomic force microscopy comes of age. Biol Cell 102: 133–143. 20001971 10.1042/BC20090127

[13] Sokolov I (2007) Atomic Force Microscopy in Cancer Cell Research. Chapter 1, 1–17. American Scientific Publishers' Inc.

[14] Cross SE, Jin YS, Rao J, Gimzewski JK (2007) Nanomechanical analysis of cells from cancer patients. Nat Nanotechnol 2: 780–783. doi: 10.1038/nnano.2007.388 18654431 10.1038/nnano.2007.388

[15] Lekka M (2012) Atomic force microscopy: A tip for diagnosing cancer. Nat Nanotechnol 7: 691–692. doi: 10.1038/nnano.2012.196 23132222 10.1038/nnano.2012.196

[16] Kaul-Ghanekar R, Singh S, Mamgain H, Jalota-Badhwar A, Paknikar KM, Chattopadhyay S (2009) Tumor suppressor protein SMAR1 modulates the roughness of cell surface: combined AFM and SEM study. BMC Cancer 9: 350 doi: 10.1186/1471-2407-9-350 19799771 10.1186/1471-2407-9-350 PMC2765988

[17] Bligh EG, Dyer WJ (1959) A rapid method of total lipid extraction and purification. Can J Biochem Physiol 37: 911–917. doi: 10.1139/o59-099 13671378 10.1139/o59-099

[18] Lara-Cruz C, Jimenez-Salazar JE, Ramon-Gallegos E, Damian-Matsumura P, Batina N (2016) Increasing roughness of the human breast cancer cell membrane through incorporation of gold nanoparticles. Int J Nanomedicine 11: 5149–5161. doi: 10.2147/IJN.S108768 27785020 10.2147/IJN.S108768 PMC5066869

[19] Francis LW, Lewis PD, Gonzalez D, Ryder TA, Webb G, Joels LA, et al (2009) Progesterone induces nano-scale molecular modifications on endometrial epithelial cell surfaces. Biol Cell 101: 481–493. doi: 10.1042/BC20080189 19236310 10.1042/BC20080189

[20] Corsetto PA, Montorfano G, Zava S, Jovenitti IE, Cremona A, Berra B, et al (2011) Effects of n-3 PUFAs on breast cancer cells through their incorporation in plasma membrane. Lipids Health Dis 10:73 doi: 10.1186/1476-511X-10-73: 73–10. 21569413 10.1186/1476-511X-10-73 PMC3127786

[21] Perez-Chacon G, Astudillo AM, Balgoma D, Balboa MA, Balsinde J (2009) Control of free arachidonic acid levels by phospholipases A2 and lysophospholipid acyltransferases. Biochim Biophys Acta 1791: 1103–1113. doi: 10.1016/j.bbalip.2009.08.007 19715771 10.1016/j.bbalip.2009.08.007

[22] Perez-Chacon G, Astudillo AM, Ruiperez V, Balboa MA, Balsinde J (2010) Signaling role for lysophosphatidylcholine acyltransferase 3 in receptor-regulated arachidonic acid reacylation reactions in human monocytes. J Immunol 184: 1071–1078. doi: 10.4049/jimmunol.0902257 20018618 10.4049/jimmunol.0902257

[23] Gijon MA, Riekhof WR, Zarini S, Murphy RC, Voelker DR (2008) Lysophospholipid acyltransferases and arachidonate recycling in human neutrophils. J Biol Chem 283: 30235–30245. doi: 10.1074/jbc.M806194200 18772128 10.1074/jbc.M806194200 PMC2573059

[24] Shindou H, Hishikawa D, Nakanishi H, Harayama T, Ishii S, Taguchi R, et al (2007) A single enzyme catalyzes both platelet-activating factor production and membrane biogenesis of inflammatory cells. Cloning and characterization of acetyl-CoA:LYSO-PAF acetyltransferase. J Biol Chem 282: 6532–6539. doi: 10.1074/jbc.M609641200 17182612 10.1074/jbc.M609641200

[25] Ricci D, Braga PC (2004) Recognizing and avoiding artifacts in AFM imaging. Methods Mol Biol 242: 25–37. 14578511 10.1385/1-59259-647-9:25

[26] Yeow N, Tabor RF, Garnier G (2017) Atomic force microscopy: From red blood cells to immunohaematology. Adv Colloid Interface Sci. doi: 10.1016/j.cis.2017.05.011 28515013 10.1016/j.cis.2017.05.011

[27] Rico F, Roca-Cusachs P, Gavara N, Farre R, Rotger M, Navajas D (2005) Probing mechanical properties of living cells by atomic force microscopy with blunted pyramidal cantilever tips. Phys Rev E Stat Nonlin Soft Matter Phys 72: 021914 doi: 10.1103/PhysRevE.72.021914 16196611 10.1103/PhysRevE.72.021914

[28] Weyn B, Kalle W, Kumar-Singh S, Van ME, Tanke H, Jacob W (1998) Atomic force microscopy: influence of air drying and fixation on the morphology and viscoelasticity of cultured cells. J Microsc 189: 172–180. 9503661 10.1046/j.1365-2818.1998.00299.x

[29] Tomankova K, Kolarova H, Vujtek M, Zapletalova H (2007) Study of Cancer Cells Used Atomic Force Microscopy In: Méndez-Vilas A, Díaz J, eds. Modern research and educational topics in microscopy. Badajoz, Spain: Formatex Research Center; 2007: 23–28.

[30] Moloney M, McDonnell L, O'Shea H (2004) Atomic force microscopy of BHK-21 cells: an investigation of cell fixation techniques. Ultramicroscopy 100: 153–161. doi: 10.1016/j.ultramic.2003.12.010 15231305 10.1016/j.ultramic.2003.12.010

[31] Lara-Cruz C, Tapia-Tapia M, Gonzalez-Nunez L, Matsumura PD, Batina N (2008) Visualización de la superficie de membrana de líneas celulares de cáncer de mama por microscopía de fuerza atómoca (AFM). IX Congreso Nacional de Microscopia, Asociación Mexicana de Microscopia A.C., https://www.yumpu.com/es/document/view/14199961/41-visualizacion-de-la-superficie-de-membrana-de-lineas-celulares-

[32] Cho SJ, Quinn AS, Stromer MH, Dash S, Cho J, Taatjes DJ, et al (2002) Structure and dynamics of the fusion pore in live cells. Cell Biol Int 26: 35–42. doi: 10.1006/cbir.2001.0849 11779219 10.1006/cbir.2001.0849

[33] Layton BE, Sastry AM, Lastoskie CM, Philbert MA, Miller TJ, Sullivan KA, et al (2004) In situ imaging of mitochondrial outer-membrane pores using atomic force microscopy. Biotechniques 37: 564–3. 15517968 10.2144/04374BI01

[34] Jaureguiberry MS, Tricerri MA, Sanchez SA, Garda HA, Finarelli GS, Gonzalez MC, et al (2010) Membrane organization and regulation of cellular cholesterol homeostasis. J Membr Biol 234: 183–194. doi: 10.1007/s00232-010-9245-6 20336284 10.1007/s00232-010-9245-6 PMC2868589

[35] Peter A, Weigert C, Staiger H, Rittig K, Cegan A, Lutz P, et al (2008) Induction of stearoyl-CoA desaturase protects human arterial endothelial cells against lipotoxicity. Am J Physiol Endocrinol Metab 295: E339–E349. doi: 10.1152/ajpendo.00022.2008 18523127 10.1152/ajpendo.00022.2008

[36] Cheng YH, Wong EW, Cheng CY (2011) Cancer/testis (CT) antigens, carcinogenesis and spermatogenesis. Spermatogenesis 1: 209–220. doi: 10.4161/spmg.1.3.17990 22319669 10.4161/spmg.1.3.17990 PMC3271663

[37] Atilla-Gokcumen GE, Muro E, Relat-Goberna J, Sasse S, Bedigian A, Coughlin ML, et al (2014) Dividing cells regulate their lipid composition and localization. Cell 156: 428–439. doi: 10.1016/j.cell.2013.12.015 24462247 10.1016/j.cell.2013.12.015 PMC3909459

[38] Wang Y, Xu C, Jiang N, Zheng L, Zeng J, Qiu C, et al (2016) Quantitative analysis of the cell-surface roughness and viscoelasticity for breast cancer cells discrimination using atomic force microscopy. Scanning 38(6):558–563. doi: 10.1002/sca.21300 26750438 10.1002/sca.21300

[39] Girasole M, Pompeo G, Cricenti A, Congiu-Castellano A, Andreola F, Serafino A, et al (2007) Roughness of the plasma membrane as an independent morphological parameter to study RBCs: a quantitative atomic force microscopy investigation. Biochim Biophys Acta 1768: 1268–1276. doi: 10.1016/j.bbamem.2007.01.014 17320813 10.1016/j.bbamem.2007.01.014

[40] Girasole M, Cricenti A, Congiu-Castellano A, Fenu S, Mancini F, et al. (2003) Low Roughness Values of RBCs Membrane in Cells with Cytoskeleton Alterations. AIP Conference Proceedings 696, 467 (2003);

[41] Kronlage C, Schafer-Herte M, Boning D, Oberleithner H, Fels J (2015) Feeling for Filaments: Quantification of the Cortical Actin Web in Live Vascular Endothelium. Biophys J 109: 687–698. doi: 10.1016/j.bpj.2015.06.066 26287621 10.1016/j.bpj.2015.06.066 PMC4547164

[42] Wang J, Wan Z, Liu W, Li L, Ren L, Wang X, et al (2009) Atomic force microscope study of tumor cell membranes following treatment with anti-cancer drugs. Biosens Bioelectron 25: 721–727. doi: 10.1016/j.bios.2009.08.011 19734031 10.1016/j.bios.2009.08.011

[43] Berdyyeva T, Woodworth CD, Sokolov I (2005) Visualization of cytoskeletal elements by the atomic force microscope. Ultramicroscopy 102: 189–198. doi: 10.1016/j.ultramic.2004.09.008 15639349 10.1016/j.ultramic.2004.09.008

[44] Li M, Liu L, Xi N, Wang Y (2016) Applications of Atomic Force Microscopy in Exploring Drug Actions in Lymphoma-Targeted Therapy at the Nanoscale. BioNanoScience 6: 22–32.

